# His6, His13, and His14 residues in Aβ 1–40 peptide significantly and specifically affect oligomeric equilibria

**DOI:** 10.1038/s41598-019-45988-1

**Published:** 2019-07-01

**Authors:** Kaja Przygońska, Magdalena Pacewicz, Wiktoria Sadowska, Jarosław Poznański, Wojciech Bal, Michał Dadlez

**Affiliations:** 1 Institute of Biochemistry and Biophysics, Polish Academy of Sciences, Warsaw, Poland; 2 Department of Chemistry, University of Warsaw, Warsaw, Poland; 3 Institute of Genetics and Biotechnology, Department of Biology, University of Warsaw, Warsaw, Poland

**Keywords:** Protein aggregation, Protein aggregation

## Abstract

Oligomers of Aβ peptide are implicated as the most probable causative agent in Alzheimer’s disease. However, their structural properties remain elusive due to the dynamic and heterogeneous character of oligomeric species coexisting in solution. Nevertheless, new approaches, mainly based on mass spectrometry, provide unique access to these different structural forms. Using these methods, we previously showed that the N-terminal, non-amyloidogenic region of Aβ is involved in the network of interactions specifically stabilizing oligomers. In the present study, we identified three histidine residues as active participants in this network. Detailed knowledge of the structural features that are potentially important for oligomer-mediated neurotoxicity is a prerequisite for the rational design of oligomerization modifiers.

## Introduction

The etiology of Alzheimer’s disease (AD), a chronic, debilitating, and incurable form of dementia, has been under investigation for decades. Currently, the consensus points to oligomeric forms of the so-called Aβ peptide as the most probable main synaptotoxic and neurotoxic agent^1–4^. Growing evidence of the key role of small, soluble Aβ oligomers calls for a detailed characterization of their structure^5,6^. These studies, however, are hampered by the heterogeneity and dynamic nature of structural species arising during the transition from monomers to oligomers^7–9^. For these reasons, classic methods of structural analysis do not readily deliver detailed information, providing characterization of the structural properties of oligomers only in the most general terms (see for example Table S1 in ref.^10^). Alternative methods of structure analysis that would provide experimental insight into each of the co-existing species and their dynamic nature need to be explored.

Mass spectrometry (MS) appeared recently as the most promising new alternative, complementing classic methods in the study of oligomerization processes^11–13^. In native MS mode^14^, mass measurements enable the separation of signals originating from oligomers of different order (i.e., of different molecular mass). When coupled with ion mobility (IM-MS), the technique also allows the separation of signals from different structural variants of oligomers of the same order and measurement of their collisional cross section (Ω, Å^2^). Therefore it allows for characterization of the structural properties of each form in separation. Recently, IM-MS has appeared as a new tool for analysis of oligomerizing molecules^15,16^, along with Aβ and its variants^17,18^. When coupled with hydrogen deuterium exchange (HDX), in solution or gas phase, MS provides insight into the structural dynamics of different oligomeric forms. Combining these approaches provides a unique analytical tool for species-specific structural characterization of forms coexisting in solution^19^. This methodology may extend knowledge on the oligomeric structures despite the necessity to transfer the molecules from solution to the gas phase. Gas-phase HDX, monitored at millisecond times after gentle ionization, has been proven capable of probing solution-like conformational states of peptides, oligomers, and proteins^20–22^. Since the correspondence of in-solution and gas-phase structures cannot be granted, we have also included dynamic light scattering (DLS) analyses of in-solution oligomeric distributions. DLS is more sensitive for larger (>100 kDa) species, but it also provides information on smaller species that can be detected by MS.

We previously analyzed the IM-MS spectra of WT Aβ 1–40 and found that the population of oligomers of a given order (e.g. trimers, tetramers, pentamers, etc.) splits into two distinct structural variants, differing in their collisional cross section and, therefore, their structure^17^. Oligomers of the same order exhibit either more extended or more compact structures (see for example Fig. 2 in ref.^17^). We have also analyzed gas-phase HDX for these forms and shown increased protection from exchange in oligomers, compared to monomers^10^. Gas-phase HDX explores the involvement of side-chain exchangeable protons and not main chain amide protons, probed in classic solution HDX. Therefore, our results demonstrate the oligomer-stabilizing involvement of Aβ side-chain protons in H-bonding networks. The majority of side-chains with exchangeable protons are localized within the N-terminal 1–16 region of Aβ. The role of the N-terminus in shaping oligomeric equilibria has only started to emerge, and the H-bonding network, involving the N-terminal side-chains in oligomers, requires more detailed characterization.

**Figure 1:**
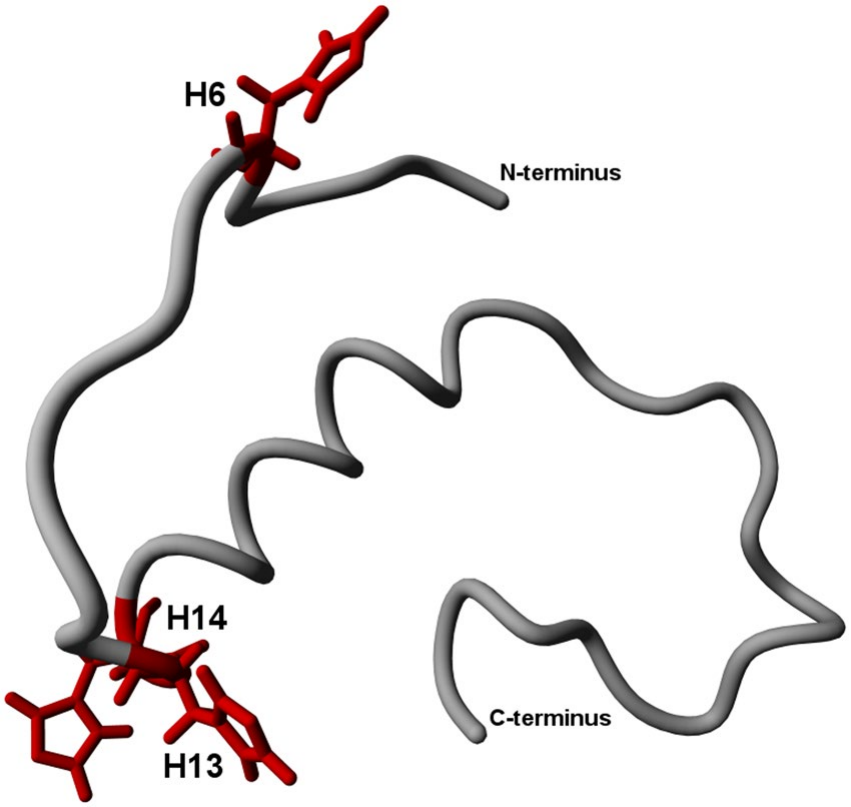
Placement of His residues within WT Aβ 1–40 sequence. Side-chain moieties of histidines are highlighted in red. PDB structure 2LFM was used.

Within the N-terminal 1–16 sequence are 12 residues with side-chains that are able to participate in H-bonding, including three histidines at positions 6, 13, and 14 (marked in Fig. 1). A notable number of studies have documented that these histidine residues influence the neurotoxic properties of the peptide^23–26^. In particular, the substitution of His14 by alanine, unlike His6 and His13, has been shown to lead to loss of toxicity^27^. Other results indicate that the peptide must be at least dimeric to exert membrane binding-mediated toxicity^28,29^.

Cu(II) binding is another putative physiological/toxic function of His moieties. Cu(II)-Aβ complexes have been proposed to participate in Aβ toxicity via redox chemistry, including generation of reactive oxygen species, and/or enhancement of aggregation and cell binding ability, including Aβ ion channel activity and membrane interactions^25,27,30–34^. AD patients exhibit abnormal accumulation of metal ions (Cu(II) and Zn(II)) in Aβ aggregates, senile plaques, and synaptic areas of the brain^35–37^. Aβ peptides self-associate faster in the presence of Cu(II) ions, with distinct morphological features. The Cu(II) ions may stabilize various structures and initiate and enhance the formation of toxic oligomers and aggregates^30,38–40^. Binding depends on pH because nearly all Cu(II) binding residues present in peptides (without Met, which does not participate in the Cu(II) binding in Aβ) are reversibly protonated, and the Cu(II) binding occurs via displacement of these protons^30,41–43^. Metal binding sites are localized in the N-terminal part of Aβ^34,44–48^. Accordingly, the structure of the binding site has been analyzed in a number of studies on the truncated versions of the peptide, such as Aβ 1–16. Interestingly, there are also lines of evidence for the formation of a Cu^2+^ complex with two Aβ molecules, which may be considered as dimeric species^49–51^.

As neurotoxicity is inherently connected to the ability of the peptide to oligomerize, mapping the determinants that define this ability is critically important. Therefore, to better understand the molecular mechanisms affecting the oligomeric distributions of Aβ 1–40 mediated by the hydrophilic N-terminal residues, we characterized the role of each of the Aβ His moieties in oligomerization. For this purpose, we characterized the structural properties of different oligomeric forms coexisting in solution in Aβ 1–40 and its three variants, H6A, H13A, and H14A, in the absence and presence of Cu(II) ions by combining dynamic light scattering (DLS) of oligomeric ensembles in solution with the MS-based analytical tools.

## Results

The aim of this work was to characterize the changes in oligomeric patterns of Aβ 1–40 upon substitution of each of histidine residue by alanine. For this purpose, we used DLS and MS-based techniques. The properties of three point mutants of peptide Aβ 1–40 (H6A, H13A, and H14A) were compared to WT Aβ 1–40. DLS measurements allowed us to characterize the oligomeric distributions directly in solution. The MS-based analyses, which allowed us to resolve signals originating from oligomers of different orders and their structural variants, included IM and HDX in the gas phase, both in the apo form and in the Cu(II)-bound form. From IM-MS measurements, the collisional cross section (Ω, Å^2^) values of different oligomeric forms could be compared, whereas application of HDX provided insight into the involvement of side-chain exchangeable protons in intramolecular or intermolecular H-bonding networks.

**Figure 2:**
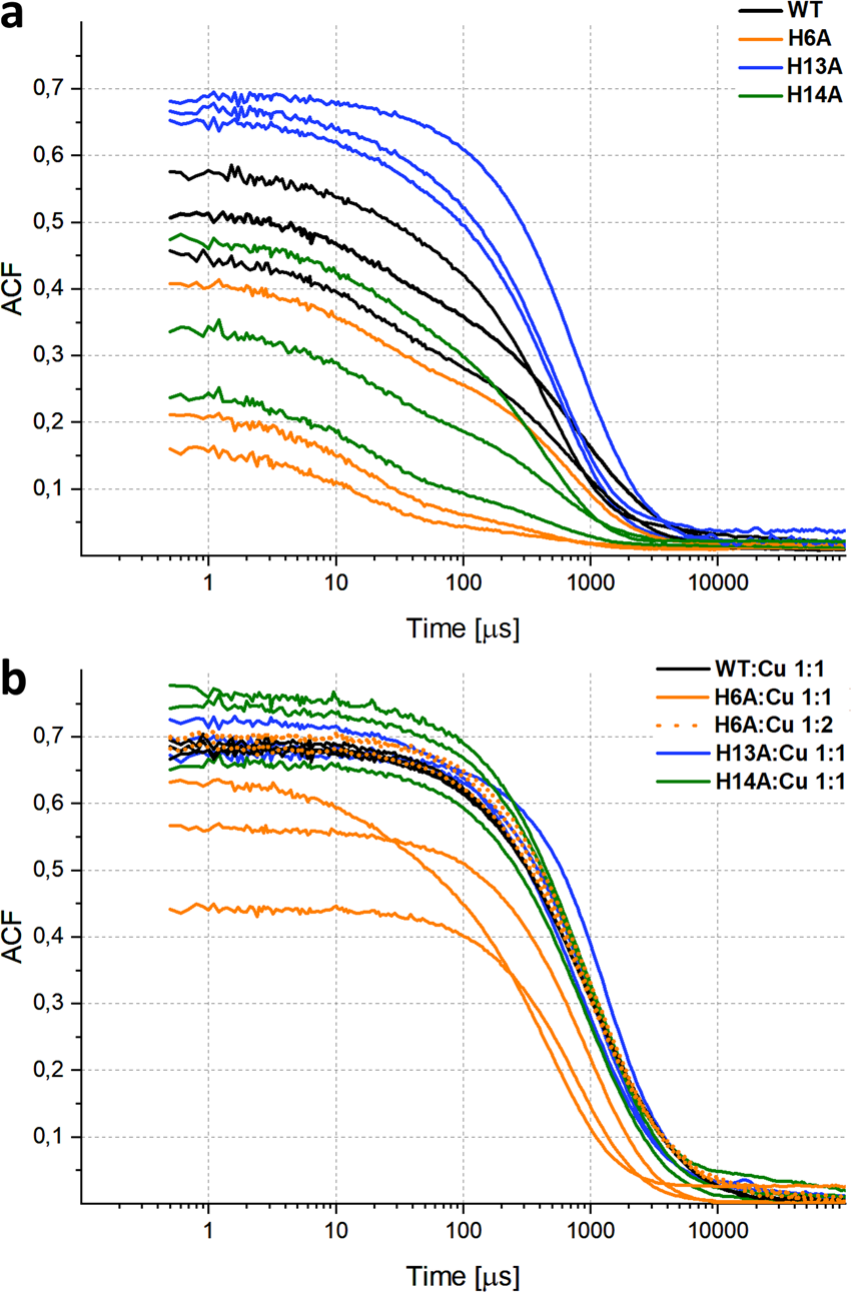
Dynamic light scattering (DLS) experiment of WT Aβ 1–40, and its three variants. Representative autocorrelation functions (ACFs) directly adapted from dynamic light scattering data obtained for WT Aβ 1–40 (black), H6A (orange), H13A (blue), and H14A (green) (**a**) and data collected in the presence of metal ion Cu(II). (**b**) Cu(II) was added in 1:1 stoichiometry, or 1:2 stoichiometry in the case of H6A (dotted orange). The ACF decays in the range of 1 down to 0, and the gap remaining to 1 observed at the shortest correlation time is indicative of the existence of monomeric or low-order oligomers, which remain undetectable. Results of three independent experiments are shown.

The results of DLS analysis of four variants of the peptide are presented in Fig. 2 in the form of the autocorrelation function (ACF) of the scattered light, which allows characterization of the distribution of oligomeric sizes. The following four parameters of the ACF curve were taken into account to interpret the results obtained in the analysis of solutions of Aβ 1–40 and its three variants: (1) the value of the signal at the shortest observable correlation time (i.e., 0.5 *μ*s (ACF0)), the smaller the fraction of monomers, the higher the intersection value of the ACF curve with the vertical axis at this correlation time; (2) the initial short-time asymptote of ACF, which reflects the presence of small oligomers; (3) the location of the midpoint of the transition (M), higher values in the time domain indicate larger aggregates; and 4) the general shape of the ACF, which reflects the homogeneity of the sample, as highly heterogeneous solutions have wide ACF transitions, whereas homogeneous distributions reveal a sharp transition.

The average values of the two parameters, ACF0 and M, for four variants with no metal added or with 1:1 Cu:peptide stoichiometry (also 1:2 in case of H6A) are shown in Table 1. In the apo form, the signal value at ACF0, at the intersection with the vertical axis, was the highest for H13A (0.67); for WT it was intermediate (0.49) and much lower for H14A (0.35) and H6A (0.26). Mutation of His to Ala at position 13 increased the fraction of oligomers compared to monomers, whereas at position 14 or 6 it decreased the oligomeric fraction. The largest impact was noted for H6A with an ACF0 of 0.26. In the case of H6A, the mixture was characterized by a very high fraction of monomers, though the reproducibility was worse with this mutant for an undetermined reason. Depending on the position, the His moiety in the WT peptide either decreased (His13) or increased (His14, His6) the fraction of monomers. It has to be stressed that the ACF0 value does not allow to measure the absolute fraction of monomeric species, for instance ACF0 of 0.5 does not imply 50% of monomers in solution. The experimental ACF function is the weighted average of ACF functions for all species in the solution, with individual weights scaled both by efficiency of scattering (proportional to diameter of an oligomer as r^6) and by concentration. So, the apparent contribution of low-order oligomers to ACF is substantially lower than that for the same number of molecules associated in high-order aggregates.

**Table 1:** The signal value at the shortest observable correlation time (ACF 0.5 *μs*) - upper row, and location of the midpoint of the transition (Midpoint ACF) – lower row, for four Aβ variants in the absence of metals and for 1:1 Cu:peptide stoichiometry (1:2 also in case of H6A).

		WT	WT 1:1 Cu	H6A	H6A 1:1 Cu	H6A 1:2 Cu	H13A	H13A 1:1 Cu	H14A	H14A 1:1 Cu
Signal value ACF at 0.5 *μs*	**AV**	**0**.**49**	**0**.**68**	**0**.**26**	**0**.**55**	**0**.**69**	**0**.**67**	**0**.**70**	**0**.**35**	**0**.**72**
	*SD*	*0*.*03*	*0*.*01*	*0*.*13*	*0*.*10*	*0*.*01*	*0*.*01*	*0*.*03*	*0*.*12*	*0*.*07*
Midpoint ACF [*μs*]	**AV**	**303**	**845**	**104**	**546**	**853**	**457**	**922**	**133**	**768**
	*SD*	*93*	*36*	*143*	*232*	*59*	*159*	*285*	*83*	*26*

The averages of three independent experiments are shown. AV, average; SD, standard deviation. The results of the statistical analysis are presented in Supplementary Information Fig. S2.

The fraction of lower-order oligomers is also reflected in the initial slope of the ACF curve. A larger slope, as observed with H6A and H14A, indicates larger populations of low-order oligomers compared to WT and H13A, where larger oligomers were preferentially populated (Fig. 2a). For H13A, this was accompanied by the narrowest distribution of oligomeric forms reflected in the sharpest transition between forms characterized by small correlation times (small oligomers) and large correlation times, representing the larger species. For WT and the two remaining variants, this distribution was broader. The replacement of His13 with Ala resulted in a more homogeneous distribution of oligomers than that observed for WT, with a decreased fraction of small oligomers. The H13A mutation also led to oligomers of an increased average size, as reflected in the M values in Table 1, whereas the other two mutants yielded smaller average oligomers. In conclusion, oligomeric distributions in solution proved to be highly sensitive to the presence or absence of single His moieties and their positions, with adverse effects observed for H13A and H6A/H14A. Our data reveal very strong and position-dependent participation of these hydrophilic, N-terminal residues in shaping the oligomeric equilibria. Though His6 critically stabilizes oligomers, the effect can be intermediate, as with His14, or even opposite, as with His13, the presence of which destabilizes oligomers in WT peptide.

Interestingly, narrower and more homogeneous distributions of ACF were induced in all four variants when Cu(II) was added in 1:1 stoichiometry (1:2 in the case of H6A) (Fig. 2b). In the presence of Cu(II), the ACF curves of WT, H13A, and H14A, and thus their oligomeric distributions, were very similar to each other (Fig. 2b) and similar to H13A in apo form. For H6A at 1:1 stoichiometry, the ACF curve differed from WT, but at 1:2 peptide:metal stoichiometry they also become similar to WT (Fig. 2b). Therefore, upon addition of metal, the fraction of oligomers increased in WT, H14A, and H6A, but for H13A it remained unchanged. For WT and H14A, the oligomerization became as efficient at 1:1 stoichiometry of Cu(II) as observed for H13A in both the apo and Cu(II)-bound form. Therefore, the oligomer-destabilizing effect of the His13 moiety, which was observed in the apo form, is removed by Cu(II) binding. On the other hand, the stabilizing effect of His14 observed in the apo form is absent in the presence of metal ion. Thus, specific oligomer-destabilizing (His13) and stabilizing (His14) interactions become substituted upon Cu(II) addition by the interactions with the metal ion. When coordinated to the Cu(II) ion, His13,14 moieties lose their ability to participate in other interactions and their impact on oligomer distribution decreases, so that oligomeric distributions in the presence of Cu(II) no longer depend on the presence of the His13 and His14 moieties. Substitution of the His6 moiety requires higher metal concentrations to become compensated. This indicates a key role of His6 as a metal-induced oligomer-ordering moiety, in agreement with its known role as the main Cu(II) binding residue^52^.

In the presence of Cu(II), the median M position was strongly shifted to longer times (WT, 845 *μ*s; H13A, 922 *μ*s; H14A, 768 *μ*s; H6A, 853 *μ*s at 1:2 stoichiometry), indicating larger average masses of Cu-bound oligomers, similar for each variant and similar to H13A apo. The transition becomes sharper in the case of all four variants, and the oligomeric distributions become more homogeneous in the presence of metal. Independent of the composition of the Cu(II)-binding moieties, metal binding enforces a more uniform, homogeneous alignment of monomers into oligomers.

**Figure 3:**
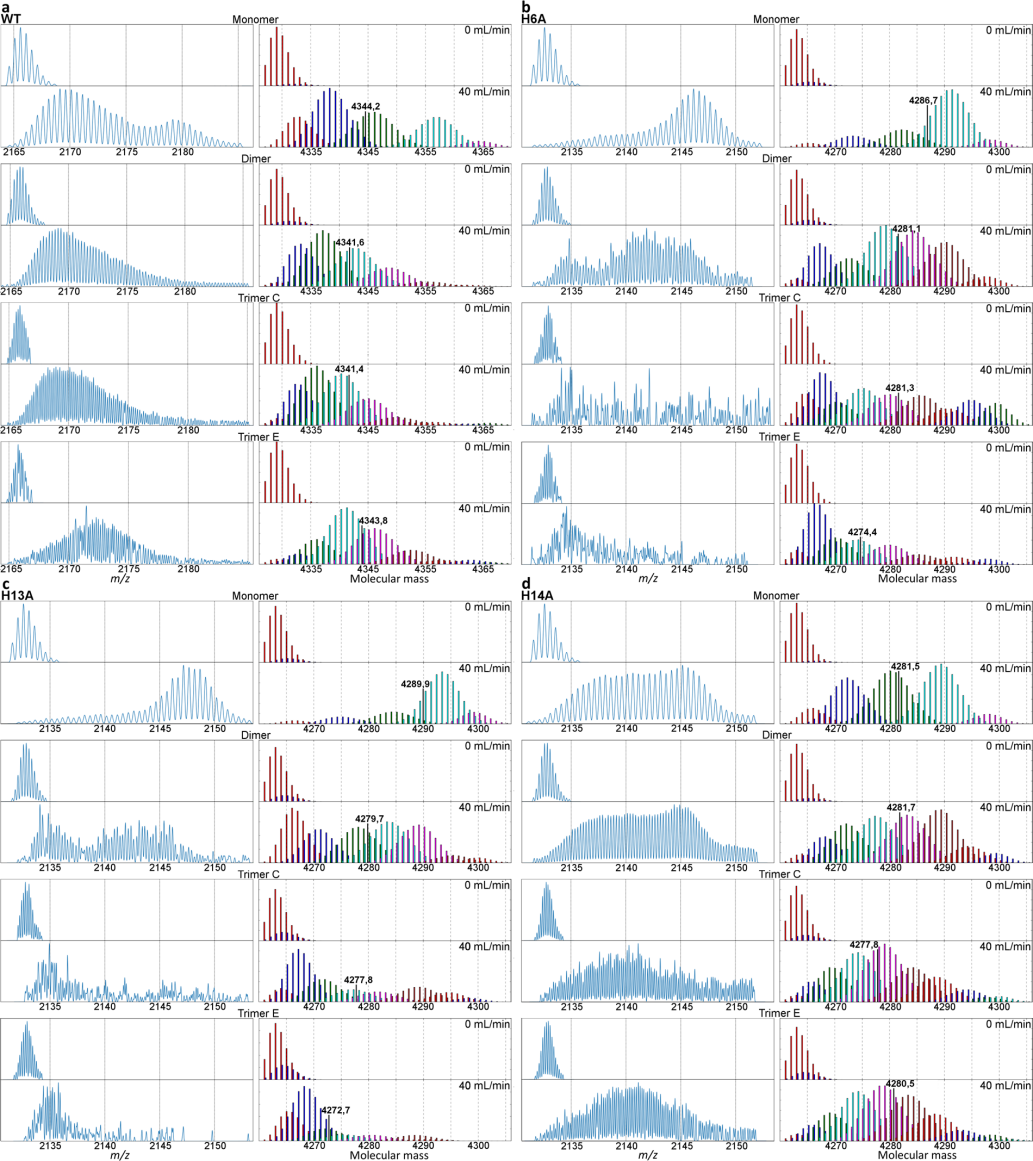
Analysis of the isotopic envelopes after gas-phase HDX-MS for selected signals of species bearing the same charge per monomer, namely MON^2+^, DIM^4+^, and TRI^6+^ of WT Aβ 1–40 (**a**) (2164–2185 m/z range), H6A (**b**), H13A (**c**), and H14A (**d**) (2130–2155 m/z range). Left panels, Isotopic envelopes corresponding to MON^2+^ (upper panels), DIM^4+^ (middle panels), and TRI^6+^ compact and extended forms (lower panels) for a make-up gas flow of 40 mL/min in the presence of ND_3_/D_2_O reagent. Right panels, Decomposition of the experimental isotopic envelope into a family of isotopic envelopes of full width at half maximum (FWHM) expected for a single conformational state. The spectra were recalculated from the *m/z* domain (left panels) to the domain of the molecular mass of a monomeric unit in an oligomer (right panels). Masses indicated in right panels are weighted average masses of all forms detected. Decomposition methodology described in the “Methods” section. Results of replicates of this experiments are shown in Supplementary Fig. S1.

Though DLS provides a more sensitive signals for relatively large (>100 kDa) oligomers it also allowed to compare the populations of low-order oligomers in the mutants. Such low-order oligomeric native-like forms can also be probed by MS-based methods, provided the measurements are carried out within milliseconds after electrospray ionization (ESI)^16,17^. Molecules at this time frame may transiently retain native-like structures in the gas phase. Here, the oligomers were subjected to gas-phase HDX at the cone exit in a submillisecond time frame after ESI, as described previously^10,53^. After the exposure of molecules to ND_3_ in the cone exit, the isotopic envelopes resulting from exchange can be analyzed. To compare the exchange between oligomers of different orders, it is necessary to limit the comparison to oligomeric states bearing the same charge per monomer due to high sensitivity of the gas phase structure to the charge state of the molecule. Therefore, exchange in MON^2+^, DIM^4+^ and TRI^6+^ forms, all bearing charge 2+ per monomer, was compared in the apo form. In the presence of Cu(II), the signal in isotopic envelopes after exchange became too weak to be analyzed. The isotopic envelopes of apopeptides after exchange obtained in three replicate experiments are shown in Fig. 3 and Supplementary Fig. S1. As noted previously^10^ for WT, in the majority of cases the isotopic envelopes after exchange were strongly split, indicating the coexistence of several structural variants for each oligomeric form at the cone exit, where the exchange takes place.

**Figure 4:**
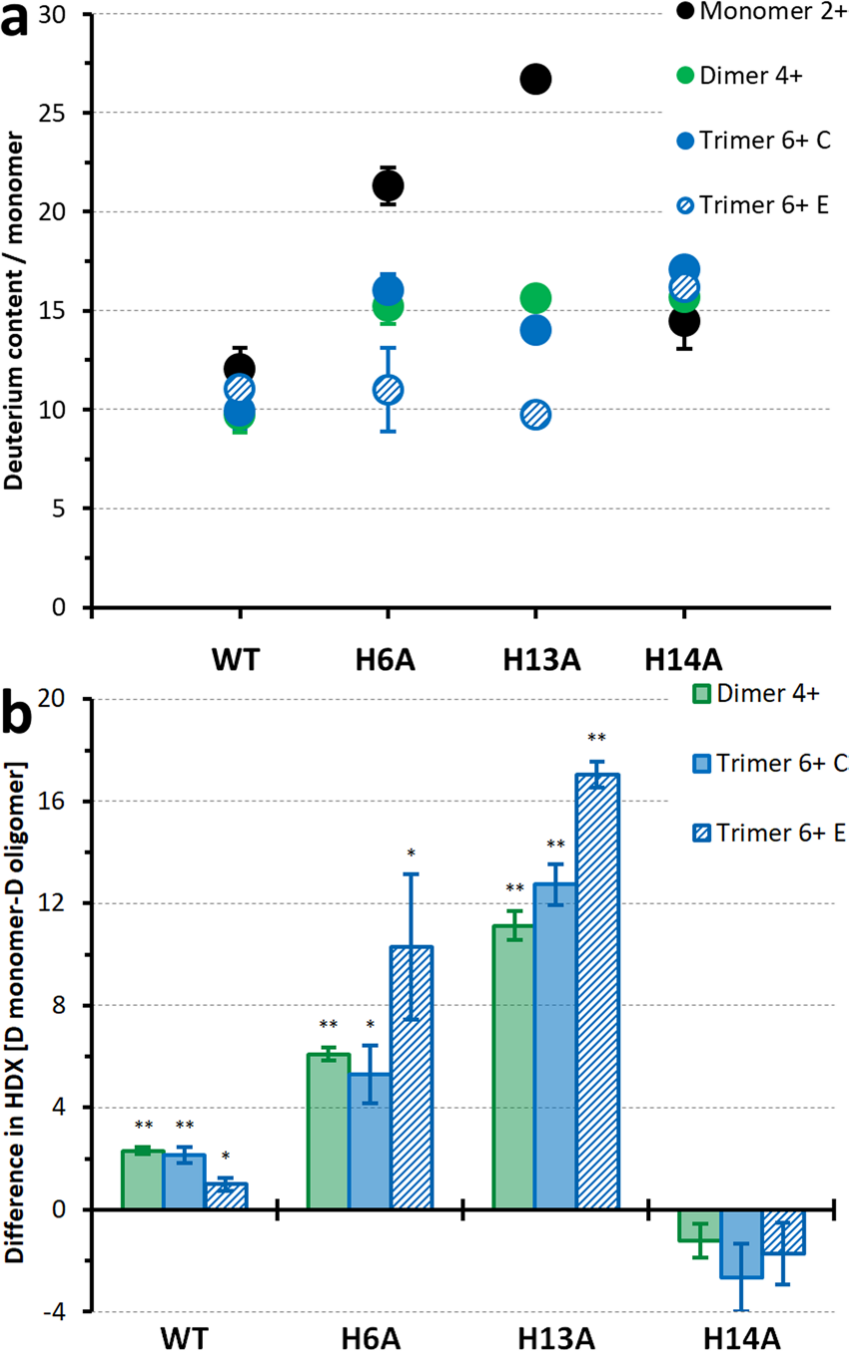
(**a**) Deuterium content per monomer (HDX/M) of individual charge states of monomer 2+ (black), dimer 4+ (green), trimer compact 6+ form (blue), and trimer 6+ extended form (dashed blue) of WT Aβ 1–40 and mutants induced by gas-phase HDX-MS at a make-up gas flow rate of 40 mL/min, averaged over all signals of the appropriate isotopic envelope. Error bars indicate the standard deviation calculated from replicate measurements (3 replicates). (**b**) Difference in deuterium uptake per monomer, as shown in (**a**), between dimers and monomers (green), trimers compact form and monomers (blue), and trimers extended form and monomers (dashed blue). The analysis is based on the signals at the *m/z* region 2164–2169 (for WT) and 2130–2155 (for mutants) which contain well-resolved isotopic envelopes from MON^2+^, DIM^4+^, and TRI^6+^ oligomeric forms bearing the same charge per monomer. The p-values were evaluated by t-test (**p-value < 0,05 or *p-value < 0,1).

In the first step of the analysis, the average masses after exchange were calculated. Inspection of average uptake levels in these forms (Fig. 4a) showed that, upon mutation, the uptake generally increases significantly compared to WT. H13A leads to the highest uptake in the monomer (26.7 Da). For H6A it was intermediate, whereas for H14A it was much lower, nearly as low as for WT (14.4 Da in H14A *vs*. 11.7 Da in WT). Substitution of His13 led to very strong destabilization of the H-bonding network in the monomer. Presence of His13 in WT and H14A is sufficient to induce substantial protection. The Aβ 1–40 molecule contains 27 non-amide heteroatom-bound protons (see Fig. 1B, C in ref.^10^) that may be exchanged during gas-phase HDX-MS, along with the charging protons. MON^2+^ contained two such charge-carrying exchangeable protons, giving a total of 29 exchangeable protons. In H13A, the MON^2+^ form underwent a nearly complete exchange. His13 and His6 seem to be the key moieties that stabilize the monomeric structure, whereas His14 is not involved much. Even in the case of monomers, the analyzed structures seem to be well defined, as the impact of His moiety removal on the overall exchange is starkly position-dependent. In other words, a single His moiety can reconfigure the structures to a high degree in a position-dependent way.

The differences in the average extent of exchange between dimers and monomers and between trimers and monomers are shown in Fig. 4b. Similar to the situation in WT, the deuterium uptake was markedly lower in H6A and H13A oligomers than in monomers. The difference was even more pronounced in these two mutants, especially H13A, than in WT. In contrast, the effect was absent with H14A, as oligomeric forms took up deuterium with the same efficiency as monomers.

The exchange for dimers remained at a similar level regardless of the variant (15.2–15.6 Da per monomer). In addition, the trimeric compact form in each variant underwent exchange to a similar extent (14.0 to 16.0 Da per monomer). In WT the exchange was on the level of 10 Da in oligomers. The removal of a His moiety deprotected oligomers (i.e., destabilized H-bonding networks) to a similar extent in each variant; thus, this effect is not position-dependent in the case of oligomers. This is in contrast to monomers, which are very strongly deprotected in H13A (mass increased by 15.0 Da compared to WT), much less in H6A (9.6 Da), and only slightly in H14A (2.7 Da).

A more detailed analysis of deuterium uptake patterns can be carried out by inspecting the isotopic envelopes (Fig. 3 and Supplementary Fig. S1). For the monomeric form of WT (see upper panel in Fig. 3a), the fast exchanging species are minor, whereas for H13A they are dominating, less so for H6A (Fig. 3b,c), and for H14A (Fig. 3d) the exchange reveals a wide distribution of states from slow to fast exchanging. A much greater fraction of faster exchanging species is present in dimeric forms of all mutants, and isotopic envelopes become much wider, with multiple forms of different levels of exchange populated. Trimeric forms of H14A also reveal much wider isotopic envelopes and the faster exchanging species than WT. For H13A and H6A trimers, the slow exchanging species seem to dominate, with even less exchange for the WT trimer compact form, accompanied by a plethora of less populated faster exchanging species. In conclusion, the effect of His substitution on exchange in apo forms is strongly position- and species-dependent. H-bonding networks involving His moieties in oligomers, but also monomers, strongly influenced the stabilities of different forms. Compared to WT, the removal of His14 decreased protection in monomers to a much smaller extent than the removal of His13. In agreement with DLS, His13 was the strongest stabilizing moiety for monomer structures.

His moieties may stabilize monomers and oligomers to a different extent, so these interactions may change their mutual population. For example, H13A seems to destabilize intra-molecular H-bonding networks in monomers to such an extent that it leads to a relative increase in the fraction of oligomers, as observed in DLS (Fig. 2a). In the other two variants (H6A and H14A), DLS revealed a decreased oligomeric fraction. For these two variants, the decreased protection of the monomeric species was much smaller, whereas decreased protection of oligomers remained the same as for H13A. For H6A and H14A, intramolecular interactions seem to be affected by the substitutions to a much smaller extent than H13A, whereas oligomer-supporting intermolecular interactions are affected to the same extent. The analysis of DLS and HDX indicates that His13 more strongly stabilizes monomers than oligomers, shifting the equilibrium in WT towards the monomeric form, as observed in DLS. For His6, the effect is the opposite, whereas His14 stabilizes oligomers but has only a weak effect on monomers, leading to an increase in the fraction of oligomers observed in DLS. His6 and His13 residues provide strong structure stabilization for monomers and oligomers. His14 has only a weak effect on monomers but stabilizes oligomers.

**Figure 5:**
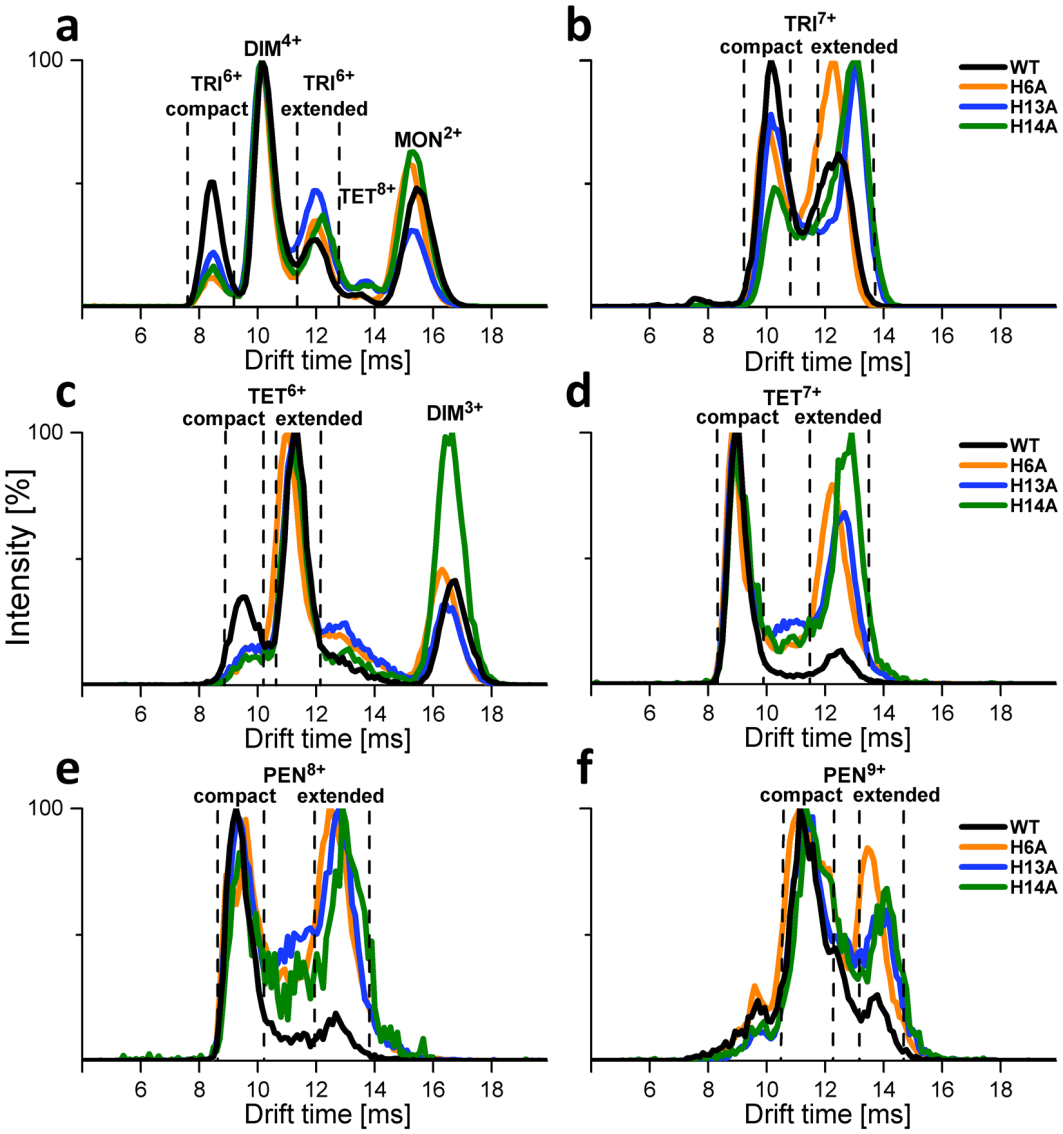
IM-MS drift time profiles of signal groups at selected regions (**a**–**f**) corresponding to WT Aβ 1–40 (black), H6A (orange), H13A (blue), and H14A (green) oligomers. For each selected *m/z* region, a profile of molecular species present at different drift times in the IM T-wave is shown, with each species assigned to a particular oligomeric charge state. These profiles represent signals that are split in the domain of the drift time, indicating the presence of multiple species, as marked. Species identification was carried out previously^17^.

All three single site substitutions of His residues to Ala led to significant changes in the IM drift time distributions compared to WT, even in the apo form. The distributions of species in the drift time domain for selected mass ranges, corresponding to different oligomeric forms, are shown in Fig. 5. The inspection of drift time profiles shows that the positions of the signals on the drift time axis (i.e., the collisional cross section Ω values of corresponding forms and, thus, their overall shapes) did not change significantly, with the exception of TRI^7+^ extended form (Fig. 5b). However, the relative population of compact and extended oligomeric forms was significantly changed in the mutants. This can be observed for TRI^6+^ and ^7+^, TET^6+^ and^7+^, and PEN^8+^ and^9+^ (Fig. 5a–f). In general, regardless of the position of His in the sequence, its absence depletes the compact form relatively more than the extended form. His residues at any position participate in the network of interactions, supporting the compact oligomeric form more than the extended form.

**Figure 6:**
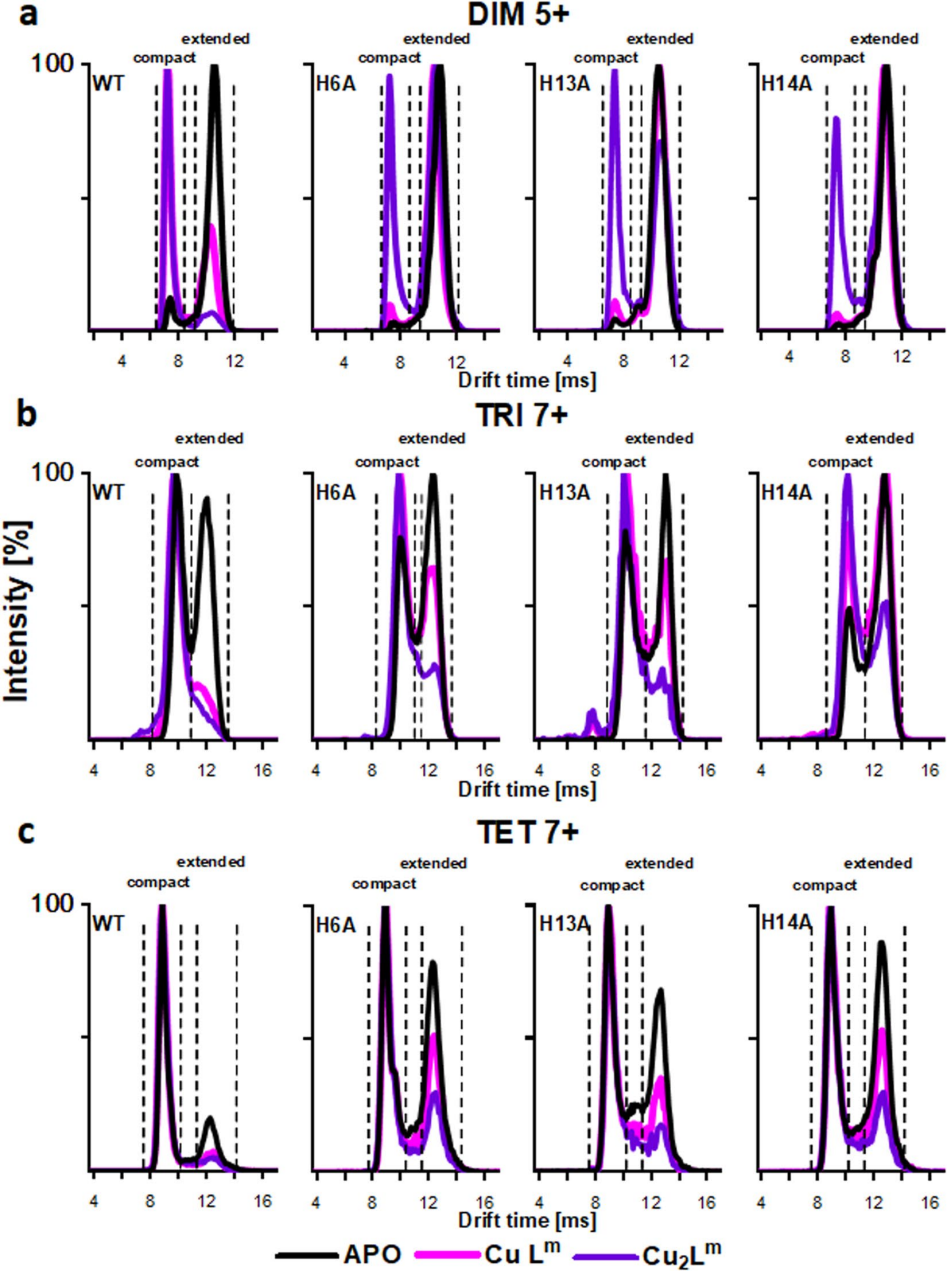
IM-MS drift time profiles of three selected regions corresponding to DIM^5+^ (**a**), TRI^7+^ (**b**) and TET^7+^ (**c**) species, extracted from spectra of WT Aβ 1–40, H6A, H13A and H14A collected in the presence of metal ions Cu(II), Cu(II) bound in complexes at different ratios (Cu_n_L^m^), where L indicates metal-liganding peptide molecule. Drift time profiles present complex of ligand-particular oligomeric charge state (L^m^) with different amounts number n of metal ions (Cu_n_), CuL^m^ (pink), Cu_2_L^m^ (purple) and compared to the control spectra collected in the absence of metals (black).

Cu(II) binding to mutants led to relative stabilization of the compact form, reverting the result of the mutation to some extent. Previously, we found that the binding of a metal ion in WT also stabilizes the compact forms, more strongly for Cu(II) than for Zn(II)^54^. However, the mutants exhibited increased populations of the extended apo form so in the mutants the Cu(II)-induced stabilization did not lead to such a strong domination of the compact form. In the cases of DIM^5+^, TRI^7+^, and TET^7+^ (Fig. 6a–c), the fraction of extended form versus compact form was much higher in the mutants than in WT. The stabilization of the compact form by Cu(II) binding, combined with the observation that this binding led to larger but more homogeneous oligomers in DLS, led to the conclusion that compact oligomeric forms are larger on average than the extended forms and presented a narrower distribution of states, especially when stabilized by metal.

## Discussion

Our results indicate that the His6, 13, and 14 moieties influence Aβ 1–40 oligomeric equilibria in a position-specific way. Their impact is much stronger in the absence of Cu(II) than in its presence. All three histidines are localized in the hydrophilic N-terminal portion of the Aβ sequence. The amyloidogenic properties thought to underlie neurotoxicity^55^ are encoded by hydrophobic residues characterized by β-structure propensities and localized mainly within the C-terminus^56^. However, N-terminally truncated peptide variant 17–40/42 (the so-called p3 peptide) is a natural product of the alternative “non-amyloidogenic” pathway of amyloid precursor protein cleavage^57^. Neurotoxic forms of Aβ peptide are expected to solely be the result of the “amyloidogenic” pathway. For this reason, p3 is thought to be benign^56,58^, though its role was not studied in depth^59^. If p3 is non-neurotoxic, then the presence of the N-terminus critically influences different aspects of oligomer toxicity, which may include pathologic oligomerization. The importance of the N-terminal part of Aβ was also verified by the analysis of mutations in the Aβ gene, which may cause familial AD (e.g., A2V, H6R-English, D7N-Tottori, D7H-Taiwanese) or be preventive (A2T)^60^. In addition, the position of residues involved in metal ion binding is localized in the N-terminal sequence and seems to be a critical agent shaping the Aβ fibrillogenic properties^61,62^. The present study complements our previous work^10^ in which we showed sequence-specific involvement of the N-terminal residues in the H-bonding network of oligomer-stabilizing interactions. We have also proposed a molecular mechanism by which this network of interactions may be crucial for the effective evolution of oligomers into higher-order structures. In the present study, we defined three residues that actively participate in this network. We have also shown that this impact is strongly position-dependent to the extent that, in the absence of Cu(II), His14 increases the population of oligomers in Aβ, whereas His13 decreases it.

The involvement of the N-terminus in the oligomer structure was previously noted in a classic HDX study that found a significant protection of N-terminal amides (res. 1–11) (see Fig. 8 in ref.^63^) in oligomeric species. The N-terminal region has been found to not be ordered in the majority of fibril structures^64^, but ordered N-terminal residues have also been reported^65^. The presence of “order” in this region was accompanied by substantial exchange at a set of N-terminal amides, contrasting with strong protection of the C-terminus, representing the core of the amyloid. Therefore, the N-terminus was ordered but more dynamic than the core region. His13 and His14 side-chain protons may also be protected from exchange in fibrils^66^.

Differences in the abundance of small oligomers between the three His to Ala variants in the 1–42 version were also observed previously by PICUP^27^. Combined with di-tyrosine formation analysis, membrane binding tests and the non-toxicity of H14A to primary cortical neurons allowed authors to conclude that His14 is critical for neurotoxicity due to a combination of reduced membrane-binding ability and less efficient di-tyrosine formation. On the other hand, Hung *et al*. showed that neurotoxicity correlates with the lipid-binding efficiency of dimers and trimers, but not monomers. Therefore, oligomerization seems to precede toxicity; oligomers have to be formed first, before the toxic effect can be revealed^28^. Interactions that destabilize or stabilize oligomers also influence neurotoxicity, though not necessarily directly.

The intriguing finding of the present study was that Cu(II) binding largely diminished the differences in oligomer distribution between WT, H13A, and H14A and led to a more uniform set of larger species, independent of the presence or absence of a His residue at either of these two positions. Histidine residues serve as metal coordination sites. In monomers, the binding modes are well characterized; Cu(II) is known to be coordinated at a neutral pH to three nitrogen atoms (3 N complex). The major component includes the N-terminal amine, the His6 imidazole, and one of the two remaining imidazoles, His13 or His14. Alternative binding modes may engage the N-terminal amine, the neighboring peptide amide, and one of the His imidazoles. In the case of monomers, which are assumed to be structurally heterogeneous and dynamic, the imidazole donors are interchangeable. Therefore, a missing moiety at position 13 or 14 may be readily substituted by the remaining coordination imidazole. Molecular models of Cu(II) binding to oligomeric structures (Fig. 6 in ref.^54^) suggest that the binding modes in which two His moieties juxtaposed in the neighboring monomers align in a parallel fashion provide imidazoles for alternative inter-monomeric coordination of a single Cu(II) ion. In H13A, complexes engaging two neighboring His14 moieties are possible, whereas in H14A the coordination via two His13A is still possible. In agreement with the structural models in which the chains in oligomers run parallel, these coordination modes of Cu(II) ions would stabilize oligomeric forms with a strict in-register parallel ordering of chains, regardless of the involvement of the His13 or His14 moieties, or both. As a result, structures become similar after Cu(II) ion binding. In addition, the Cu(II) binding by His13 in WT and H14A, engaging the His13 residue, releases the oligomer-destabilizing interactions of His13, and the differences observed between H13A and WT and H14A in terms of the oligomer population of their apo forms are no longer observed upon Cu(II) binding. This provides an additional reason for the observed similarity in oligomer distributions and their increased homogeneity in WT, H13A, and H14A in the presence of divalent copper. Our results indicate a unique role of Cu(II) binding as an oligomer-ordering mechanism.

In conclusion, His residues have a strong, position-dependent impact on oligomer populations. Not only the hydrophobic amyloidogenic side-chains are important for the structural evolution of oligomeric forms, but also other interactions, including the N-terminal hydrophilic residues.

## Methods

General. MS and DLS experiments were carried out essentially as described previously^10^. Description of these experiments is reproduced here.

### Aβ variant expression

Aβ 1–40 peptide and its variants were obtained by expression in *Escherichia coli* and purified using HPLC as described previously^67^. Peptide variants were obtained by site-directed mutagenesis (Stratagene Kit). Aβ peptide mutants have changes at positions H6A, H13A, and H14A. The identity and purity of each molecule was verified using a Q-ToF Premier ESI-MS instrument (Waters). Typically, the concentration of the peptide in the stock solution was 100 *μ*M and the pH adjusted to 7.4 with ammonia. The freshly purified Abeta1–40 WT and its variants after HPLC were used in all types of analysis. All experiments were carried out immediately after the LC purifications step and mass and concentration measurements. ThT binding experiments carried out using stock solutions (Supplementary Fig. S4) showed that the experiments were completed within the lag phase of fibril formation so in the absence of fibrils. NaOH, HCl, Tris, and 26% ammonia-D_3_ in D_2_O 99.5% (ND_3_/D_2_O) were purchased from Merck (Darmstadt, Germany) and ammonium acetate from Fluka (the Netherlands).

### Cu(II) experiments

Copper(II) acetate was purchased from Sigma-Aldrich Chemical Co. Metal solutions were prepared from weighed amounts using analysis grade water (Baker) to obtain concentrations of 9.4 mM (Cu). The concentration of Cu(CH_3_COO)_2_ was verified using the Lambert–Beer law (*A = εlc*) with a molar extinction coefficient value for Cu^2+^_aq_ of 12.0 M^−1^ cm^−1^ at λ = 810 nm^68^.

### Ion mobility mass spectrometry

Experiments were performed using a hybrid Q-TOF mass spectrometer with ion mobility capabilities (Synapt G2 HDMS; Waters Corp., Wilmslow, UK). Samples of Aβ 1–40 WT, H6A, H13A, and H14A at 100 *μ*M in 10 mM CH_3_COONH_4_ (pH 7.4, when necessary the pH was adjusted with ammonia) with or without addition of Cu(CH_3_COO)_2_ were infused directly at 7 *μ*L/min into the ion source of a mass spectrometer using a glass Hamilton syringe through a stainless steel capillary. The mass signals were measured in the 400–4000 *m/z* range at the rate of 1 scan per second. The analyzed spectra were the average of 200 scans. The instrument was tuned to obtain the best possible signal and HDX efficiency using the electrospray positive ion mode with a capillary voltage of 2.8 kV and a sample cone voltage of 37 V. The source and desolvation temperatures were maintained at 85 °C and 180 °C, respectively. The mobility T-wave cell was operated at a pressure of 2.5 mbar of nitrogen, with a wave velocity of 300 m/s and amplitude (T-wave height) of 40 V. Data acquisition and processing were carried out with the MassLynx V4.1 (Waters) and DriftScope V2.1 (Waters) software supplied with the instrument. Each analysis of drift time profiles for Aβ WT, H6A, H13A, and H14A was carried out under the same experimental conditions. All data were repeated for batch-to-batch replicates (n = 3 or more) to confirm the reproducibility of the results.

### Gas-phase HDX-MS

Gas-phase HDX-MS was performed in the ion source region of a commercially available Synapt G2 HDMS instrument immediately downstream of the primary cone exit (sample cone) as described elsewhere^53^. Briefly, 2.0 ml of aqueous ND_3_/D_2_O reagent was added to the standard ETD reagent vial with the ETD reagent removed. To control reagent flow, the mass spectrometer software was set in ETD-mode with a HDX reagent gas flow rate of 0–50 mL/min (N_2_ gas); 50 mL/min is the maximum setting on the Synapt G2 HDMS instrument. N_2_ gas was passed through the headspace of the reagent pot and through a needle (corona discharge needle) downstream of the primary cone exit. The trap T-Wave wave height was set to +6.0 V to prevent unwanted electron transfer reactions in the case of residual ETD reagent in the tubing. Control experiments were also performed under identical conditions with the only difference being the presence of non-deuterated NH_3_/H_2_O instead of ND_3_. This procedure allowed us to test whether the presence of a basic gas would affect the experiment, and no changes in ionization, adduct formation, or conformation were observed.

### Data analysis

Processing of all mass spectra was carried out using MassLynx V4.1 software with a Savitzky-Golay smoothing function (3, 5) and subsequent centering of the peaks. The deuterium content of peptides was determined using Excel 2013 (Microsoft Corp., Redmond, WA, USA) by calculating the difference in the intensity-weighted centroid average masses of deuterated ions with respect to those from a non-deuterated control sample recorded in the absence of ND_3_ gas. All data were derived from at least three or more replicate measurements.

An in-house procedure was developed for deconvolution of the complex, split isotopic envelopes corresponding to the differently exchanging species. First, the procedure allowed us to calculate the mass distributions expected at each stage of exchange for a single uniform conformation of a peptide of a given mass and number of exchangeable protons. The calculation was carried out using a simplifying assumption of equal probability of exchange for each exchangeable proton. If the probability was not equal (which is highly likely), the distributions necessarily became narrower. Thus, the result of this assumption is that we could model the widest possible distribution expected for a single state. As a result, the number of detected states may only be higher, but cannot be lower. Using this approach, we thus identified the minimum number of states present during exchange that would account for the experimental distributions. As a result of the first step a set of 200 theoretical uniformly deuterated single-state distributions ranging from 0% to 100% deuterium uptake was simulated for each sample. For the deconvolution procedure, both centrioided experimental distributions and theoretical distributions were represented as 500-element vectors, calculated by convolution with a Gaussian function and sampling at points uniformly distributed along the specified mass range of theoretical isotopic envelopes. The resulting linear equations were solved using boosted Gold algorithm as described in ref.^69^. with 10000 iterations, 100 boosting steps and p = 1.2. This allowed us to obtain a linear combination of single-state distributions that fits the measured distribution while meeting physical constraints (small number of non-zero elements, no negative elements). In case of two neighboring non-zero elements in the solution vector, only a single component distribution was reported in the results, with deuterium uptake linearly interpolated between theoretical uptakes corresponding to these elements.

### Dynamic light scattering

All DLS experiments were carried out at 25 °C with a DynaPro NanoStar apparatus (Wyatt Technology, Santa Barbara, CA, USA) equipped with a 661 nm laser and the 90 detection angle. The ACF for the light scattered by Aβ WT, H6A, H13A, and H14A (at 100 *μ*M in 10 mM CH_3_COONH_4_, pH 7.4) with or without addition of Cu(CH_3_COO)_2_, solution placed in an Eppendorf UVette disposable cuvette (50–2000 *μ*L) was measured and further analyzed in the range of 0.5 *μ*s to 0.2 s using Dynamics software (Wyatt Technology, ver. 7.0.2.7). All samples were filtered with 0.45-*μ*m pore syringe filter and additionally centrifuged (9000 g) for 3 minutes directly before the measurement. For each sample, a series of at least three successive repetitions, 50 acquisitions of 10 s each, were collected, and these 10 s accumulations with abnormally high SOS function and/or highly fluctuating SLS signals were removed from further analysis. As no apparent time trend in SLS data were observed, the DLS data collected upon the first repetition (i.e., during the initial 10 min after sample dilution) were averaged and further analyzed.

### Statistical analysis

All statistical analyses were performed using R package (version 3.4.1.; The R Foundation for Statistical Computing, Vienna, Austria). Fisher F-test was applied to asses identity of ACF function recorded for individual peptides. The ratio of residual variance was analysed according to F-test. Since for each peptide three independent replicates were analysed, every pair of peptides leads to six ACF curves from six independent experiments. H0 hypothesis claims that all six experiments originate from the same oligomeric distribution, which is represented by the average of six analysed ACF curves. The alternative hypothesis (H1) claims that six curves originate from two different distributions represented by two average ACF functions calculated separately for each variant. Fisher F-test estimates the probability of that these residual variances are equal, i.e. H0 hypothesis cannot be rejected. Residual variances were analysed with 5 N and 4 N degrees of freedom, respectively, where N = 266 is a number of data-points recorded in the range of 0.5 *μ*s to 0.1 s. Results shown in Fig. 4b were subjected to t-test analysis of the differences between exchange in monomers and oligomers, testing the H0 hypothesis that the difference is zero.

### Thioflavin T fluorescence assay

Commercially available Thioflavin T (ThT) was purchased from Sigma Aldrich and a stock solution of concentration 2,5 mM in Mili-Q water was prepared and stored the solution at −18 °C. Freshly received stock samples of Aβ 1–40 WT, H6A, H13A, and H14A in 10 mM CH_3_COONH_4_ (pH 7.4, when necessary the pH was adjusted with ammonia) were prepared to perform the fluorescence study, appropriate volume of peptide sample was taken out from the stock solution and was mixed with 10 μL of ThT solution (2,5 mM); to accomplish final peptide and ThT concentration of 100 *μ*M, final volume was made up to 250 *μ*L in 10 mM CH_3_COONH_4_. For ThT fluorescence assay, emission was measured from 450 nm to 650 nm and excitation at 440 nm, using a slit of 1 nm on a FP-6500 spectrofluorometer instrument (Jasco, Tokyo, Japan). The content of the cuvettes was pipetted up and down before every measurement. The fluorescence was measured at various time points, up to 72 h after the final SpeedVac purification step. From the machine text file was taken and graphs were plotted using OriginPro software (version 8, OriginLab Corporation, Massachusetts, USA).

## Supplementary information

Supplementary Information

## Data Availability

All data generated or analyzed during this study are included in this published article.

## References

[1] Hardy J (2006). Has the amyloid cascade hypothesis for Alzheimer’s disease been proved?. Curr. Alzheimer Res..

[2] Haass C, Selkoe DJ (2007). Soluble protein oligomers in neurodegeneration: lessons from the Alzheimer’s amyloid beta-peptide. Nat. Rev. Mol. Cell Biol..

[3] Benilova I, Karran E, De Strooper B (2012). The toxic Aβ oligomer and Alzheimer’s disease: an emperor in need of clothes. Nat. Neurosci..

[4] Viola KL, Klein WL (2015). Amyloid β oligomers in Alzheimer’s disease pathogenesis, treatment, and diagnosis. Acta Neuropathol..

[5] Klyubin I, Cullen WK, Hu NW, Rowan MJ (2012). Alzheimer’s disease Aβ assemblies mediating rapid disruption of synaptic plasticity and memory. Mol. Brain.

[6] Larson ME, Lesné SE (2012). Soluble Aβ oligomer production and toxicity. J. Neurochem..

[7] Roychaudhuri R, Yang M, Hoshi MM, Teplow DB (2009). Amyloid β-protein assembly and Alzheimer disease. J. Biol. Chem..

[8] Rangachari V (2007). Amyloid-β(1-42) rapidly forms protofibrils and oligomers by distinct pathways in low concentrations of sodium dodecylsulfate. Biochemistry.

[9] Sarkar B (2014). Significant structural differences between transient amyloid-β oligomers and less-toxic fibrils in regions known to harbor familial Alzheimer’s mutations. Angew. Chem. Int. Ed. Engl..

[10] Przygońska K, Poznański J, Mistarz UH, Rand KD, Dadlez M (2018). Side-chain moieties from the N-terminal region of Aβ are Involved in an oligomer-stabilizing network of interactions. PLoS One.

[11] Woods LA, Radford SE, Ashcroft AE (2013). Advances in ion mobility spectrometry-mass spectrometry reveal key insights into amyloid assembly. Biochim. Biophys. Acta.

[12] Rajabi K, Ashcroft AE, Radford SE (2015). Mass spectrometric methods to analyze the structural organization of macromolecular complexes. Methods.

[13] Young LM, Ashcroft AE, Radford SE (2017). Small molecule probes of protein aggregation. Curr. Opin. Chem. Biol..

[14] Leney AC, Heck AJR (2017). Native Mass Spectrometry: What is in the Name?. J. Am. Soc. Mass Spectrom..

[15] Ashcroft AE (2010). Mass spectrometry and the amyloid problem–how far can we go in the gas phase?. J. Am. Soc. Mass Spectrom..

[16] Smith AM, Jahn TR, Ashcroft AE, Radford SE (2006). Direct observation of oligomeric species formed in the early stages of amyloid fibril formation using electrospray ionisation mass spectrometry. J. Mol. Biol..

[17] Kłoniecki M (2011). Ion mobility separation coupled with MS detects two structural states of Alzheimer’s disease Aβ1–40 peptide oligomers. J. Mol. Biol..

[18] Bernstein SL (2009). Amyloid-β protein oligomerization and the importance of tetramers and dodecamers in the aetiology of Alzheimer’s disease. Nat. Chem..

[19] Beeston HS, Ault JR, Pringle SD, Brown JM, Ashcroft AE (2015). Changes in protein structure monitored by use of gas-phase hydrogen/deuterium exchange. Proteomics.

[20] Badman ER, Hoaglund-Hyzer CS, Clemmer DE (2001). Monitoring structural changes of proteins in an ion trap over approximately 10–200 ms: unfolding transitions in cytochrome c ions. Anal. Chem..

[21] Breuker K, McLafferty FW (2008). Stepwise evolution of protein native structure with electrospray into the gas phase, 10(−12) to 10(2) s. Proc. Natl. Acad. Sci. USA.

[22] Wyttenbach T, Bowers MT (2011). Structural stability from solution to the gas phase: native solution structure of ubiquitin survives analysis in a solvent-free ion mobility-mass spectrometry environment. J. Phys. Chem. B.

[23] Liu S-T, Howlett G, Barrow CJ (1999). Histidine-13 Is a Crucial Residue in the Zinc Ion-Induced Aggregation of the Aβ Peptide of Alzheimer’s Disease^†^. Biochemistry.

[24] Arispe N, Diaz JC, Flora M (2008). Efficiency of Histidine-Associating Compounds for Blocking the Alzheimer’s Aβ Channel Activity and Cytotoxicity. Biophys. J..

[25] Tickler AK (2005). Methylation of the imidazole side chains of the Alzheimer disease amyloid-β peptide results in abolition of superoxide dismutase-like structures and inhibition of neurotoxicity. J. Biol. Chem..

[26] Smith DP (2006). Copper-mediated amyloid-β toxicity is associated with an intermolecular histidine bridge. J. Biol. Chem..

[27] Smith DG (2010). Histidine 14 modulates membrane binding and neurotoxicity of the Alzheimer’s disease amyloid-β peptide. J. Alzheimer’s Dis..

[28] Hung, L. W. *et al*. Amyloid-Peptide (A) Neurotoxicity Is Modulated by the Rate of Peptide Aggregation: A Dimers and Trimers Correlate with Neurotoxicity, 10.1523/JNEUROSCI.3916-08.2008 (2008). 10.1523/JNEUROSCI.3916-08.2008 PMC6671645 19005060

[29] Ciccotosto GD (2011). Stereospecific interactions are necessary for Alzheimer disease amyloid-β toxicity. Neurobiol. Aging.

[30] Faller P, Hureau C, Berthoumieu O (2013). Role of Metal Ions in the Self-assembly of the Alzheimer’s Amyloid-β Peptide. Inorg. Chem..

[31] Pedersen JT (2015). Aggregation-Prone Amyloid-β Cu(II) Species Formed on the Millisecond Timescale under Mildly Acidic Conditions. ChemBioChem.

[32] Cheignon C (2018). Oxidative stress and the amyloid beta peptide in Alzheimer’s disease. Redox Biol..

[33] Atrián-Blasco, E., Del Barrio, M., Faller, P. & Hureau, C. Ascorbate Oxidation by Cu(Amyloid-β) Complexes: Determination of the Intrinsic Rate as a Function of Alterations in the Peptide Sequence Revealing Key Residues for Reactive Oxygen Species Production, 10.1021/acs.analchem.8b00740 (2018). 10.1021/acs.analchem.8b00740 PMC6120677 29611698

[34] Atrián-Blasco E (2018). Cu and Zn coordination to amyloid peptides: From fascinating chemistry to debated pathological relevance. Coord. Chem. Rev..

[35] Lovell MA, Robertson JD, Teesdale WJ, Campbell JL, Markesbery WR (1998). Copper, iron and zinc in Alzheimer’s disease senile plaques. J. Neurol. Sci..

[36] Cater MA (2008). Intracellular copper deficiency increases amyloid-beta secretion by diverse mechanisms. Biochem. J..

[37] Bush AI (2013). The metal theory of Alzheimer’s disease. J. Alzheimers. Dis..

[38] Atwood CS (2000). Characterization of copper interactions with Alzheimer amyloid β peptides: Identification of an attomolar-affinity copper binding site on amyloid β1–42. J. Neurochem..

[39] Drew SC, Masters CL, Barnham KJ (2010). Alzheimer’s Aβ Peptides with Disease-Associated N-Terminal Modifications: Influence of Isomerisation, Truncation and Mutation on Cu2+ Coordination. PLoS One.

[40] Sarkar B, Das AK, Maiti S (2013). Thermodynamically stable amyloid-β monomers have much lower membrane affinity than the small oligomers. Front. Physiol..

[41] Syme CD, Nadal RC, Rigby SEJ, Viles JH (2004). Copper binding to the amyloid-beta (Abeta) peptide associated with Alzheimer’s disease: folding, coordination geometry, pH dependence, stoichiometry, and affinity of Abeta-(1-28): insights from a range of complementary spectroscopic techniques. J. Biol. Chem..

[42] Kozłowski H, Bal W, Dyba M, Kowalik-Jankowska T (1999). Specific structure–stability relations in metallopeptides. Coord. Chem. Rev..

[43] Sigel H, Martin RB (1982). Coordinating properties of the amide bond. Stability and structure of metal ion complexes of peptides and related ligands. Chem. Rev..

[44] Streltsov V (2008). X-ray absorption and diffraction studies of the metal binding sites in amyloid β-peptide. Eur. Biophys. J..

[45] Drew SC, Barnham KJ (2011). The Heterogeneous Nature of Cu 2þ Interactions with Alzheimer’s Amyloid-β Peptide. Acc. Chem. Res..

[46] Minicozzi V (2008). Identifying the minimal copper- and zinc-binding site sequence in amyloid-beta peptides. J. Biol. Chem..

[47] Hane F, Tran G, Attwood SJ, Leonenko Z (2013). Cu2+ Affects Amyloid-β (1–42) Aggregation by Increasing Peptide-Peptide Binding Forces. PLoS One.

[48] Conte-Daban, A. *et al*. Link between Affinity and Cu(II) Binding Sites to Amyloid-β Peptides Evaluated by a New Water-Soluble UV-Visible Ratiometric Dye with a Moderate Cu(II) Affinity, 10.1021/acs.analchem.6b04979. 10.1021/acs.analchem.6b04979 PMC5714188 28208266

[49] Pedersen JT (2011). Rapid Formation of a Preoligomeric Peptide-Metal-Peptide Complex Following Copper(II) Binding to Amyloid β Peptides. Angew. Chemie Int. Ed..

[50] Branch T, Barahona M, Dodson CA, Ying L (2017). Kinetic Analysis Reveals the Identity of Aβ-Metal Complex Responsible for the Initial Aggregation of Aβ in the Synapse. ACS Chem. Neurosci..

[51] Goch W, Bal W (2017). Numerical Simulations Reveal Randomness of Cu(II) Induced Aβ Peptide Dimerization under Conditions Present in Glutamatergic Synapses. PLoS One.

[52] Cheignon C (2017). Identification of key structural features of the elusive Cu-Aβ complex that generates ROS in Alzheimer’s disease. Chem. Sci..

[53] Mistarz UH, Brown JM, Haselmann KF, Rand KD (2014). A Simple Setup for Gas-Phase H/D Exchange Mass Spectrometry Coupled to Electron Transfer Dissociation and Ion Mobility for Analysis of Polypeptide Structure on a Liquid Chromatographic Timescale. Anal. Chem..

[54] Sitkiewicz E, Kłoniecki M, Poznański J, Bal W, Dadlez M (2014). Factors influencing compact-extended structure equilibrium in oligomers of aβ1–40 peptide - An ion mobility mass spectrometry study. J. Mol. Biol..

[55] Hunter S, Brayne C (2012). Relationships between the amyloid precursor protein and its various proteolytic fragments and neuronal systems. Alzheimers. Res. Ther..

[56] Walsh DM (2002). Naturally secreted oligomers of amyloid beta protein potently inhibit hippocampal long-term potentiation *in vivo*. Nature.

[57] Higgins LS, Murphy GM, Forno LS, Catalano R, Cordell B (1996). P3 beta-amyloid peptide has a unique and potentially pathogenic immunohistochemical profile in Alzheimer’s disease brain. Am. J. Pathol..

[58] Checler F (1995). Processing of the beta-amyloid precursor protein and its regulation in Alzheimer’s disease. J. Neurochem..

[59] Kotler SA (2015). High-resolution NMR characterization of low abundance oligomers of amyloid-β without purification. Sci. Rep..

[60] Jonsson T (2012). A mutation in APP protects against Alzheimer’s disease and age-related cognitive decline. Nature.

[61] Olofsson A, Lindhagen-Persson M, Vestling M, Sauer-Eriksson AE, Ohman A (2009). Quenched hydrogen/deuterium exchange NMR characterization of amyloid-beta peptide aggregates formed in the presence of Cu2+ or Zn2+. FEBS J..

[62] Dong J (2007). Engineering metal ion coordination to regulate amyloid fibril assembly and toxicity. Proc. Natl. Acad. Sci. USA.

[63] Gremer L (2017). Fibril structure of amyloid-β(1–42) by cryo–electron microscopy. Science (80-.)..

[64] Kheterpal I, Chen M, Cook KD, Wetzel R (2006). Structural differences in Aβ amyloid protofibrils and fibrils mapped by hydrogen exchange-mass spectrometry with on-line proteolytic fragmentation. J. Mol. Biol..

[65] Lu JX (2013). Molecular structure of β-amyloid fibrils in Alzheimer’s disease brain tissue. Cell.

[66] Agarwal V (2013). Hydrogen bonding involving side chain exchangeable groups stabilizes amyloid quarternary structure. Phys. Chem. Chem. Phys..

[67] Rózga M, Kłoniecki M, Jabłonowska A, Dadlez M, Bal W (2007). The binding constant for amyloid Aβ40 peptide interaction with human serum albumin. Biochem. Biophys. Res. Commun..

[68] Prenesti E, Berto S (2002). Interaction of copper(II) with imidazole pyridine nitrogen-containing ligands in aqueous medium: a spectroscopic study. J. Inorg. Biochem..

[69] Morháč M, Matoušek V (2011). High-resolution boosted deconvolution of spectroscopic data. J. Comput. Appl. Math..

