# Supplementary material for: His6, His13, and His14 residues in Aβ 1–40 peptide significantly and specifically affect oligomeric equilibria
Source: Sci Rep. 2019 Jul 1;9:9449. doi: 10.1038/s41598-019-45988-1 (PMC6602940; doi:10.1038/s41598-019-45988-1)
Supplement: Supplementary file 1 — Supplementary Information [file 41598_2019_45988_MOESM1_ESM.pdf]

Supplementary Information

His6, His13, and His14 residues in Aβ 1-40 peptide significantly and specifically affect oligomeric equilibria.

Kaja Przygońska[1], Magdalena Pacewicz[1], Wiktoria Sadowska[1,2], Jarosław Poznański[1], Wojciech Bal[1], and Michał Dadlez[1,3]

1: Institute of Biochemistry and Biophysics, Polish Academy of Sciences, Warsaw, Poland
2: Department of Chemistry, University of Warsaw, Warsaw, Poland
3: Institute of Genetics and Biotechnology, Department of Biology, University of Warsaw, Warsaw, Poland

To whom correspondence should be addressed: Prof. Michał Dadlez, Institute of Biochemistry and Biophysics, Polish Academy of Sciences, Pawińskiego 5a Street, 02-106 Warsaw, Poland; Telephone: +48 22 592 34 71; FAX: +48 22 658 47 66; E-mail: michald@ibb.waw.pl

Figure S1.

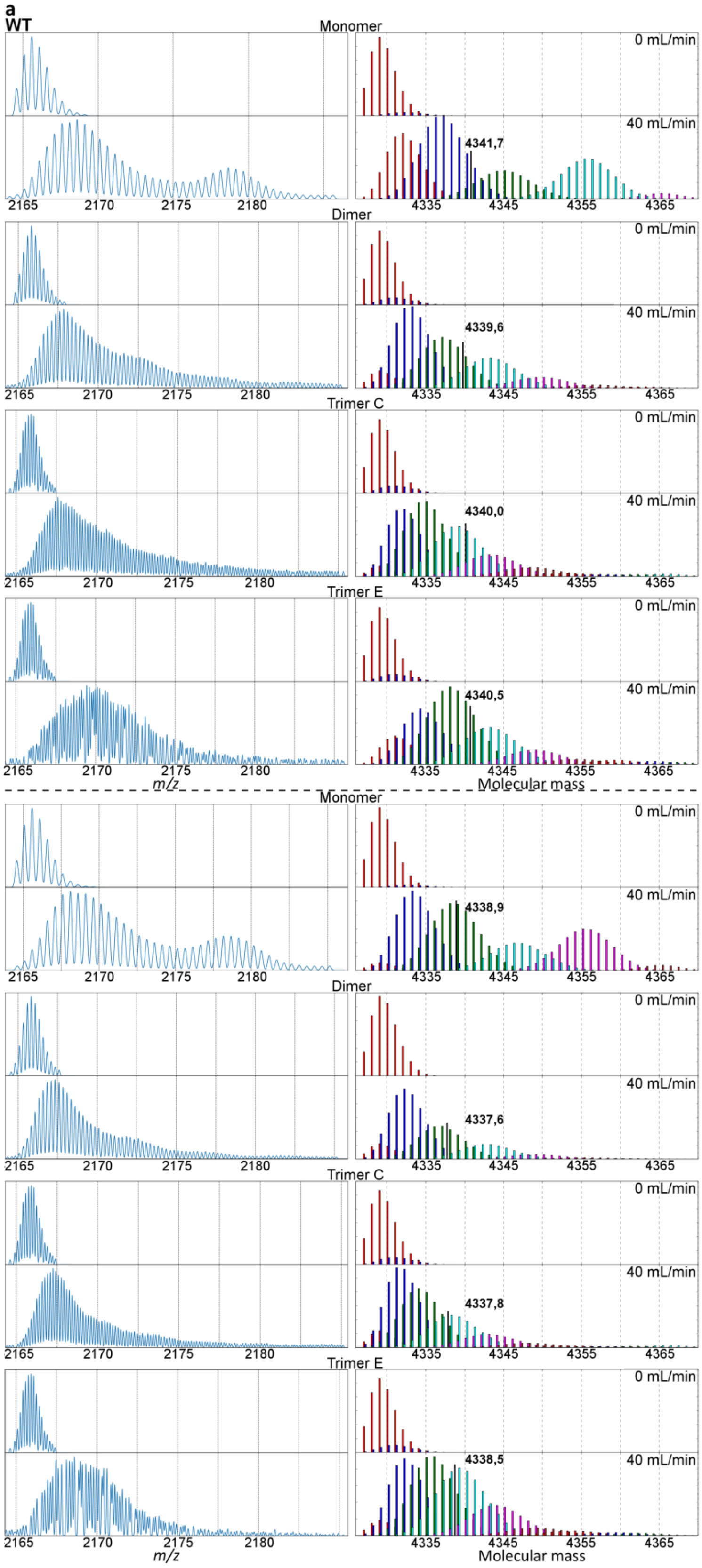

a
WT
Monomer
0 mL/min
40 mL/min
4341,7
Dimer
4339,6
Trimer C
4340,0
Trimer E
4340,5
m/z
Molecular mass
Monomer
4338,9
Dimer
4337,6
Trimer C
4337,8
Trimer E
4338,5

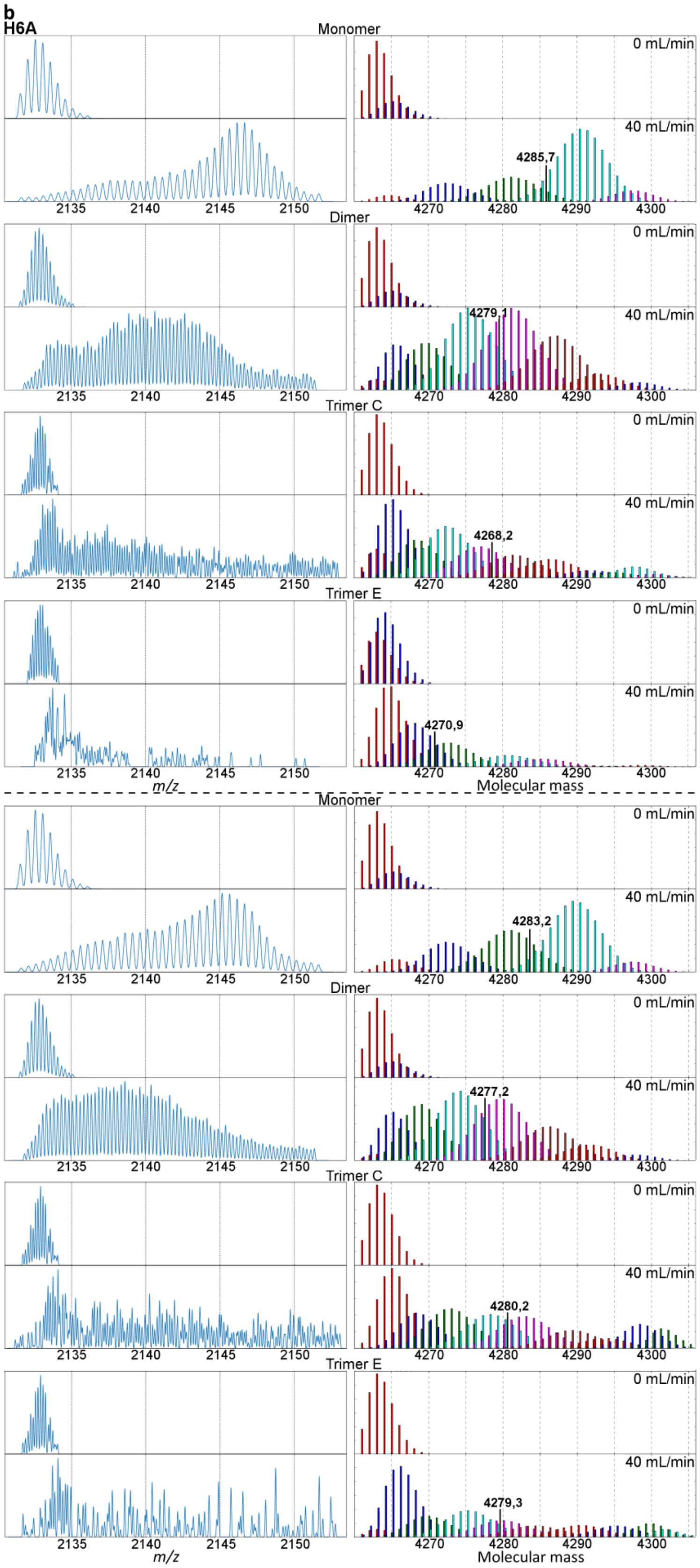
b
H6A
Monomer
0 mL/min
40 mL/min
4285,7
Dimer
4279,1
Trimer C
4268,2
Trimer E
4270,9
m/z
Molecular mass
Monomer
4283,2
Dimer
4277,2
Trimer C
4280,2
Trimer E
4279,3
2135
2140
2145
2150
4270
4280
4290
4300

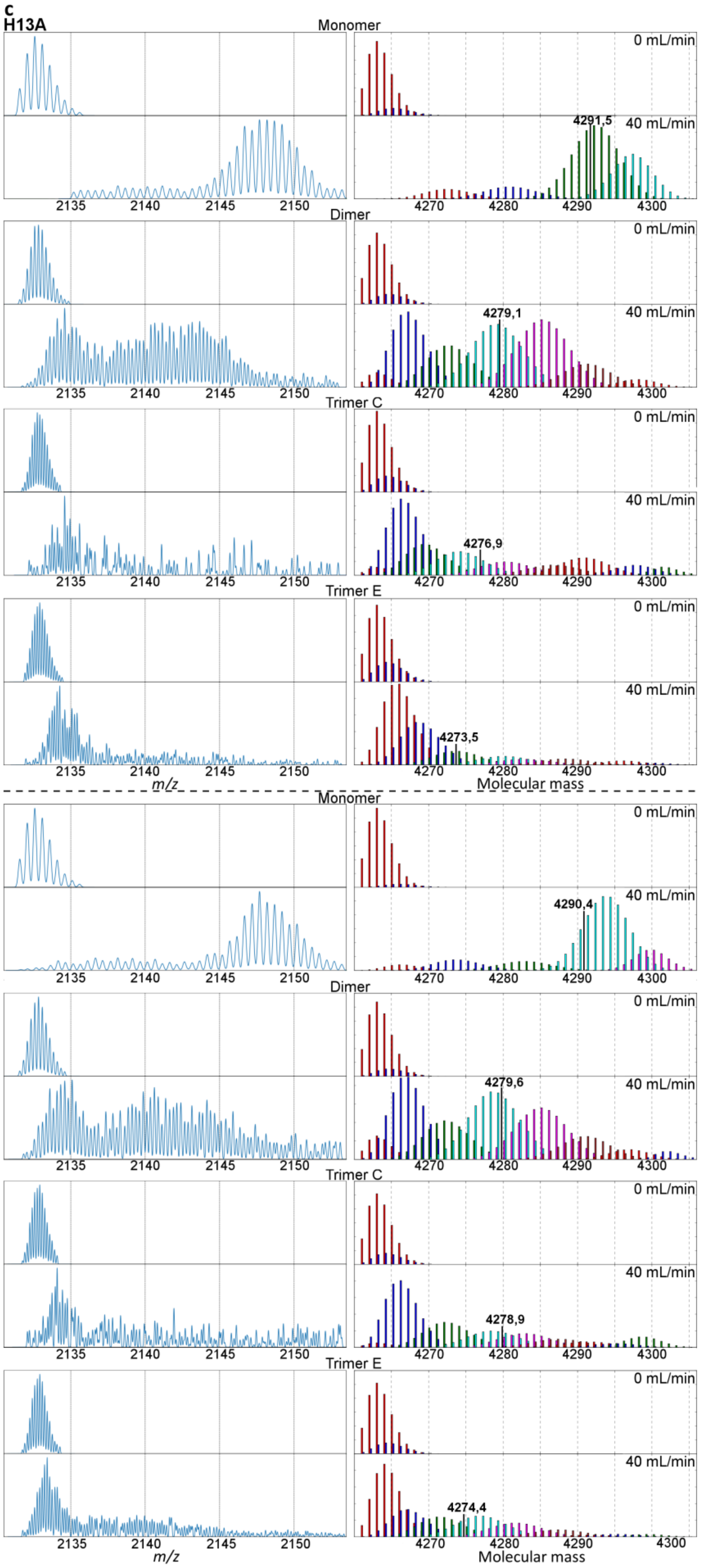
c
H13A
Monomer
0 mL/min
40 mL/min
4291,5
Dimer
4279,1
Trimer C
4276,9
Trimer E
4273,5
2135
2140
2145
2150
4270
4280
4290
4300
m/z
Molecular mass
Monomer
4290,4
Dimer
4279,6
Trimer C
4278,9
Trimer E
4274,4

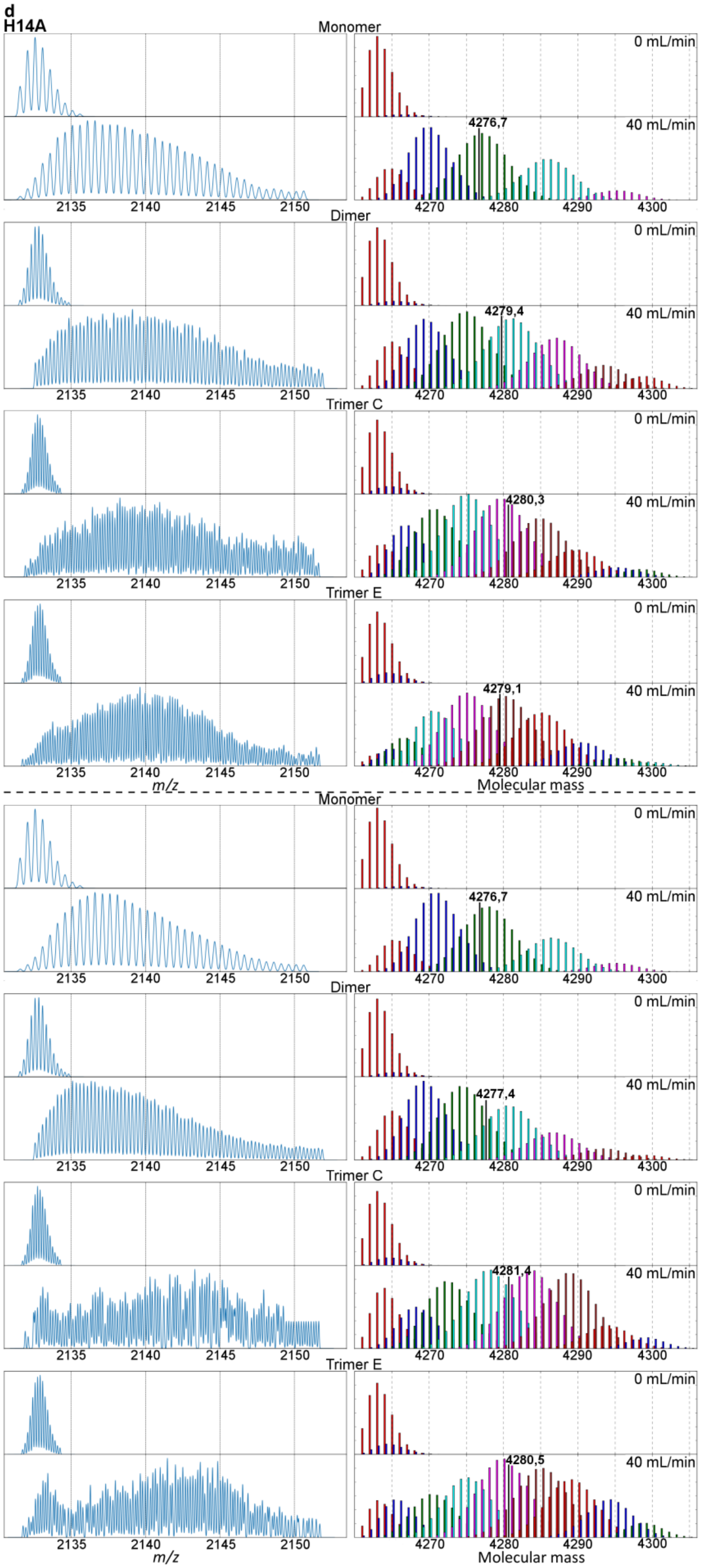
d
H14A
Monomer
0 mL/min
40 mL/min
4276,7
Dimer
4279,4
Trimer C
4280,3
Trimer E
4279,1
2135
2140
2145
2150
4270
4280
4290
4300
m/z
Molecular mass
Monomer
4276,7
Dimer
4277,4
Trimer C
4281,4
Trimer E
4280,5

Figure S1. Analysis of the isotopic envelopes after gas-phase HDX-MS for selected signals of species bearing the same charge per monomer, namely MON^{2+}, DIM^{4+}, and TRI^{6+} compact and extended forms (2164-2185 or 2130-2155 m/z range). Two subsequent replicates (separated by a dashed line) of WT Aβ 1-40 (a), H6A (b), H13A (c), and H14A (d) are compared. Left panels, Isotopic envelopes corresponding to MON^{2+} (upper panels), DIM^{4+} (upper middle panels), TRI^{6+} compact form (lower middle panels), and TRI^{6+} extended form (lower panels) for a make-up gas flow of 40 mL/min in the presence of ND_3/D_2O reagent. Right panels, Decomposition of the experimental isotopic envelope into a family of isotopic envelopes of full width at half maximum (FWHM) expected for a single conformational state. The spectra were recalculated from the *m/z* domain (left panels) to the domain of the molecular mass of a monomeric unit in an oligomer (right panels).

Figure S2.

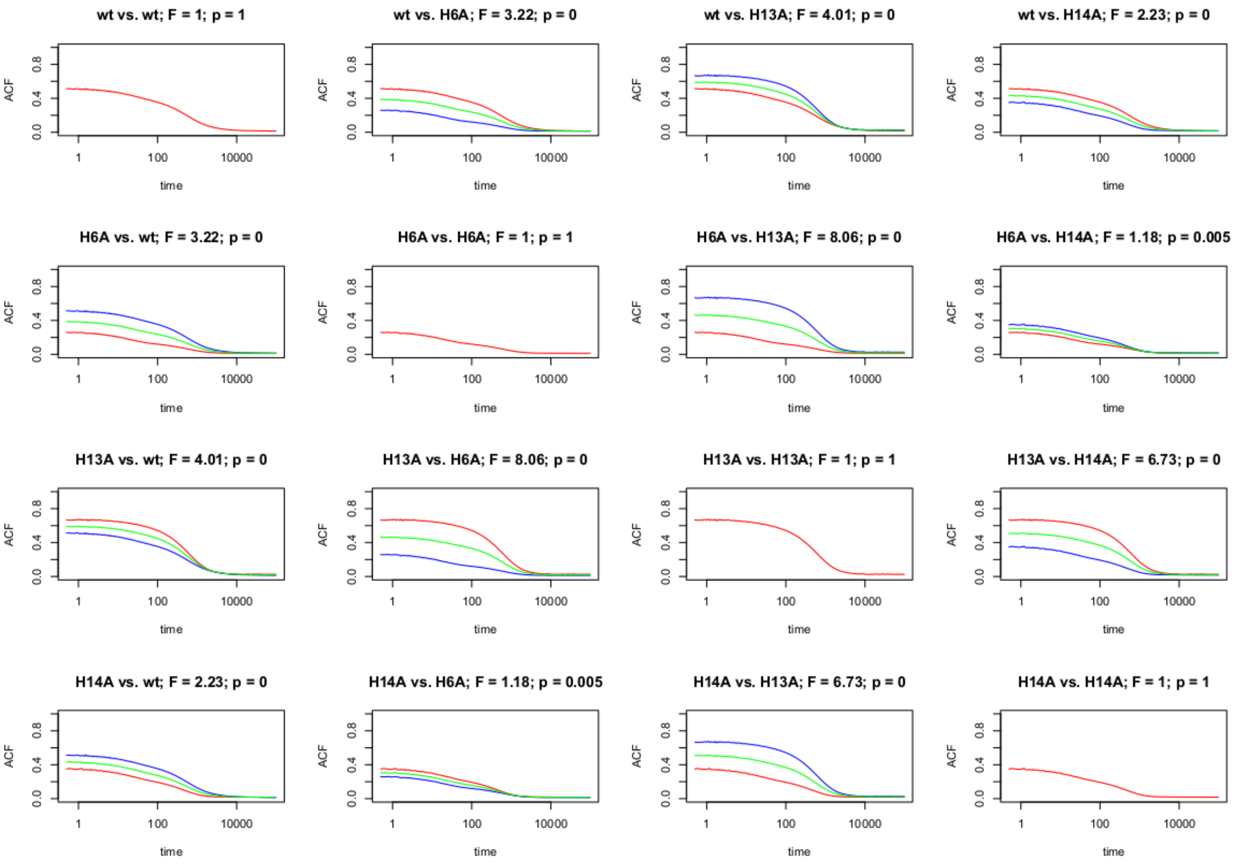

Figure S2.

Fisher F-test was applied to asses identity of ACF function recorded for individual peptides. The ratio of residual variance was analysed according to F-test with 5N and 4N degrees of freedom, respectively, where N = 266 is a number of data-points recorded in the range of 0.5 us to 0.1s. The model of two ACF functions was always found significantly better, after Bonferroni correction for multiple comparisons even for H6A vs. H14A p<0.03. The most relevant parameters of ACF curves (i.e. initial value at 0.5 μs and midpoint correlation time) are collected in Table 1. F stands for the F-test statistics and p stands for the associated p-value.

Figure S3.

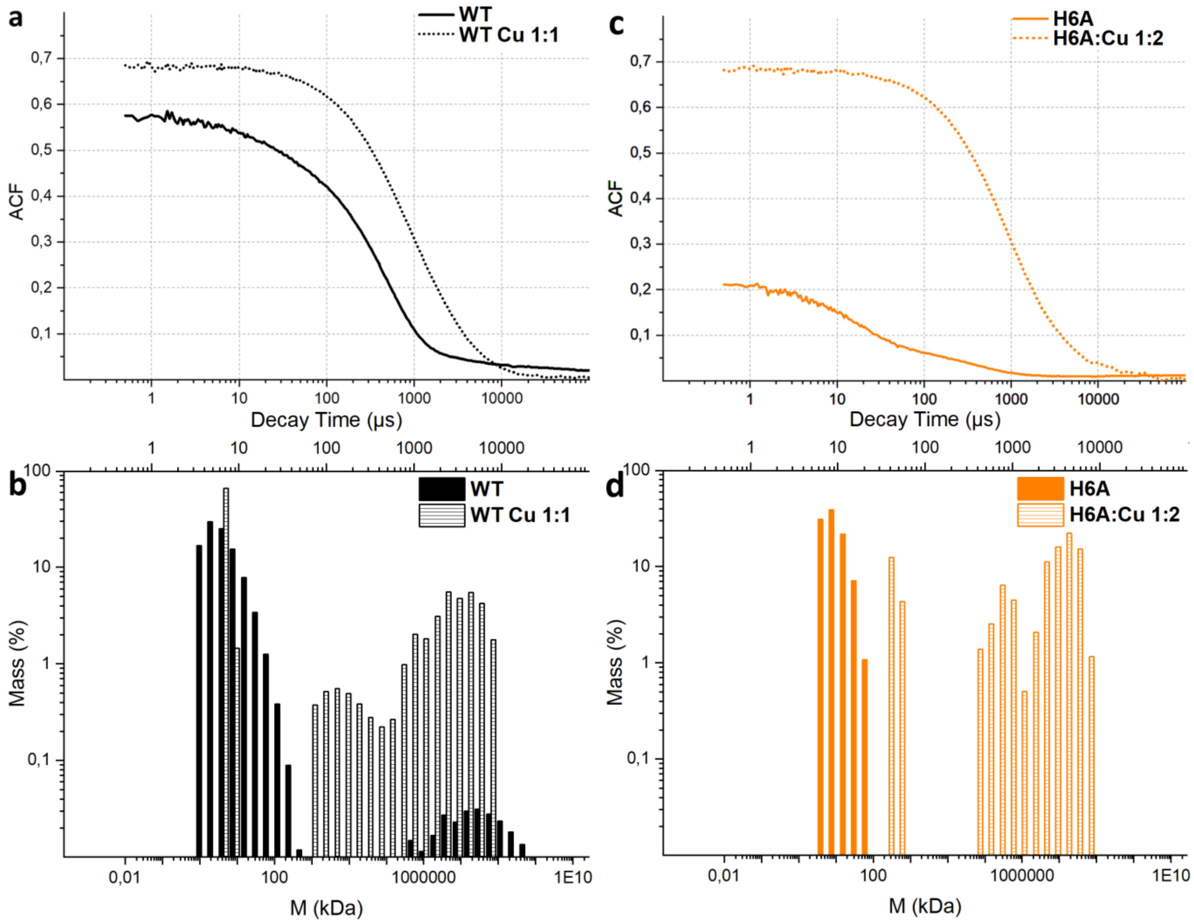
a
WT
WT Cu 1:1
ACF
Decay Time (µs)
b
WT
WT Cu 1:1
Mass (%)
M (kDa)
c
H6A
H6A:Cu 1:2
ACF
Decay Time (µs)
d
H6A
H6A:Cu 1:2
Mass (%)
M (kDa)

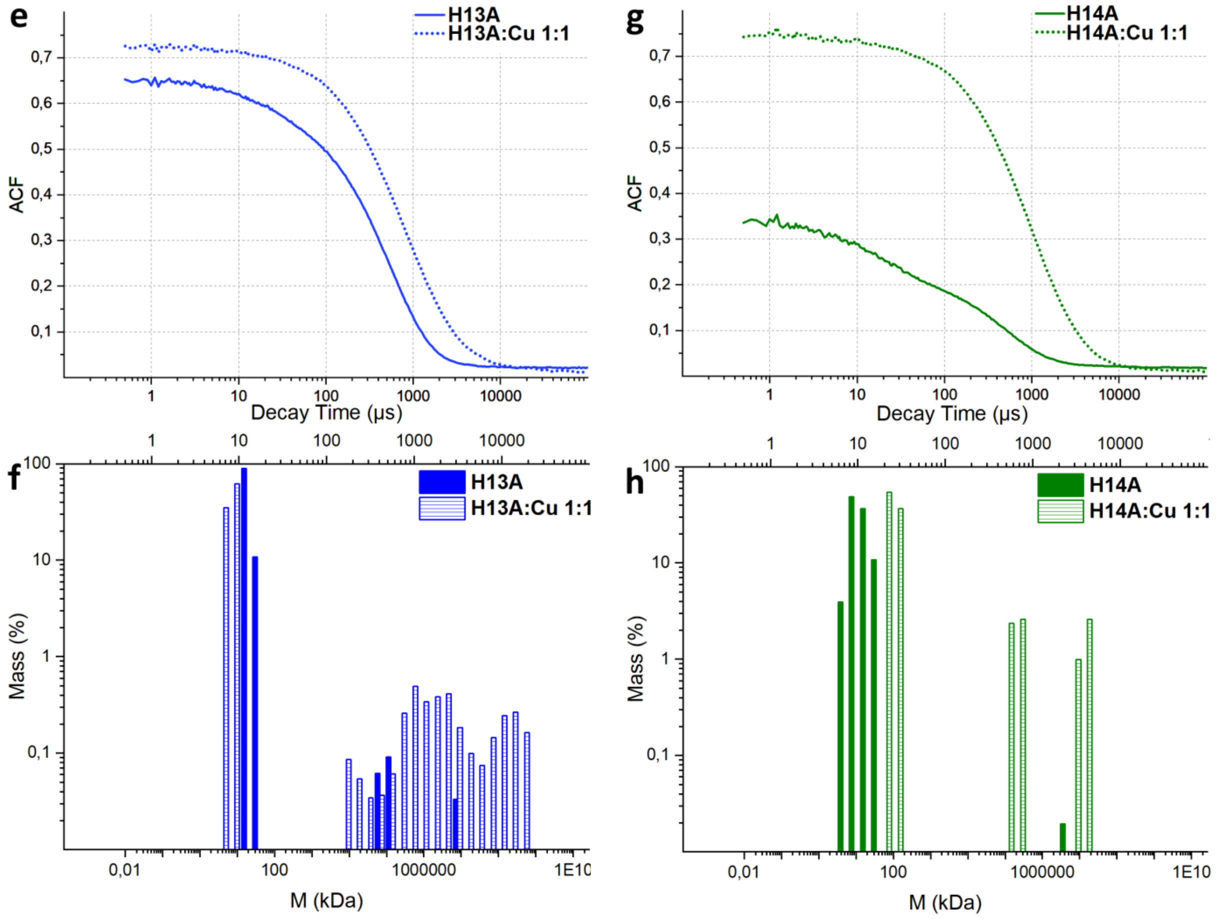

Figure S3.

Dynamic light scattering data (DLS) obtained for WT Aβ 1-40 (black), H6A (orange), H13A (blue), H14A (green) in the absence (solid) and presence (dotted) of metal ion Cu(II). ACF function estimated directly from DLS data obtained for WT Aβ 1-40 (a), H6A (c), H13A (e), H14A (g) are in parallel presented their deconvolution to the relative mass distribution (b, d, f, h).

Figure S4.

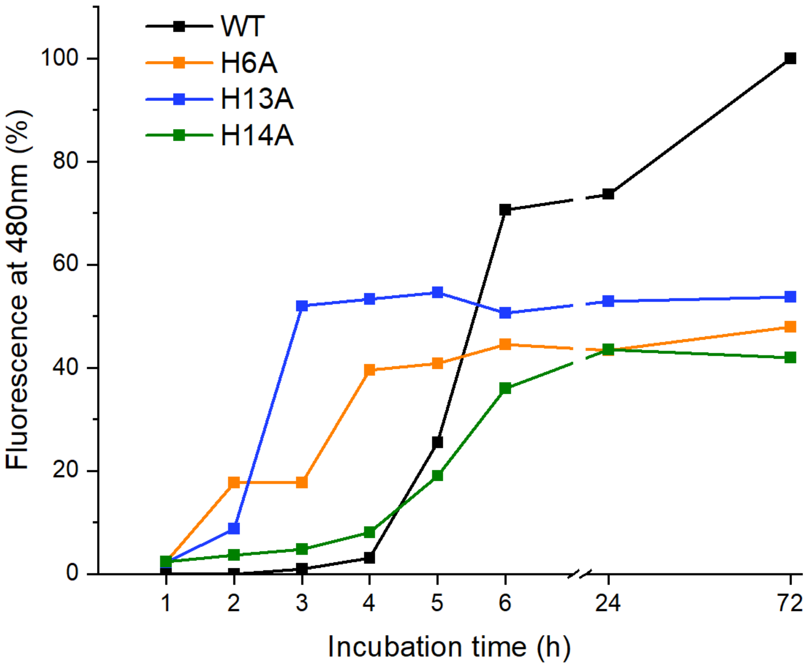

Figure S4.

ThT fluorescence assay obtained for WT Aβ 1-40 (black), H6A (orange), H13A (blue), H14A (green). Aβ variants at concentration of 100 μM in 10 mM CH_3COONH_4, pH 7.4 buffer were incubated at room temperature in the presence of 100 μM ThT. ThT binding to sheet content in samples of Aβ is expressed as fluorescence intensity at 480 nm. Data were normalized to the maximal ThT signal and represented as percentage fluorescence.
